# Pedestrian multiple-object tracking based on FairMOT and circle loss

**DOI:** 10.1038/s41598-023-31806-2

**Published:** 2023-03-20

**Authors:** Jin Che, Yuting He, Jinman Wu

**Affiliations:** 1 School of Physics and Electronic-Electrical Engineering, Ningxia University, Yinchuan, 750021 China; 2 Ningxia Key Laboratory of Intelligent Sensing for Desert Information, Ningxia University, Yinchuan, 750021 China

**Keywords:** Engineering, Electrical and electronic engineering

## Abstract

Multi-object Tracking is an important issue that has been widely investigated in computer vision. However, in practical applications, moving targets are often occluded due to complex changes in the background, which leads to frequent pedestrian ID switches in multi-object tracking. To solve the problem, we present a multi-object tracking algorithm based on FairMOT and Circle Loss. In this paper, HRNet is adopted as the baseline. Then, Polarized Self-Attention is added to HRNet-w32 to obtain weights of helpful information based on its modeling advantages. Moreover, the re-identification branch is optimized, and the Circle Loss is selected as the loss function to acquire more discriminative pedestrian features and to distinguish different pedestrians. The method proposed is assessed on the public MOT17 datasets. The experimental results show that the MOTA score achieves 69.5%, IDF1 reaches 70.0%, and the number of ID switches (IDs) decreases 636 times compared to the TraDes algorithm.

## Introduction

As an important application in the field of computer vision in recent years, multi-object tracking (MOT) has been widely used in video surveillance, urban security, automatic vehicle systems, and other fields. The main task of MOT is to locate multiple specific targets and simultaneously track and record their trajectories^1^. Nevertheless, challenges, such as highly similar target appearance, frequent target occlusion, and target number changing, will be encountered in practical applications. Hence, it has received extensive attention from many outstanding scholars worldwide, and many effective solutions have been proposed to solve these challenges. Meanwhile, as a classic direction in the field of multiple object tracking, pedestrian multi-object tracking is not only a “hot spot” in academic research but also a “big demand” in practical applications.

According to the way of target location, there are two types of multi-object tracking algorithms: tracking-by-detection (TBD) and detection-free-tracking (DFT). Although the DFT paradigm does not require a detector, it needs to manually calibrate the target in the first frame image, which is not applicable when faced with an uncertain number of pedestrians. In contrast, the TBD paradigm’s performance relies on the detector results. With the continuous optimization of the detection algorithm, the tracking performance of the TBD paradigm also improves. Therefore, many studies are focusing on the TBD paradigm to solve pedestrian multi-object tracking tasks.

Bewley et al.^2^ proposed the SORT algorithm, which combines the Kalman Filter and the Hungarian Algorithm to effectively associate objects and improve real-time tracking. Wojke et al.^3^ presented the DeepSORT algorithm, which carries over the thought of the Kalman filter combined with the Hungarian algorithm in the SORT algorithm. In addition, aiming at the problem that the SORT algorithm does not pay too much attention to the frequent identity switching caused by occlusion during the tracking process, adds a pedestrian recognition network learnt on a large-scale pedestrian re-identification dataset to reduce identity switching under the premise of ensuring real-time performance. Chen et al.^4^ proposed the MOTDT algorithm. Aiming at the misleading of tracking caused by unreliable detection results, the results of detection and tracking are combined as candidate objects, and then a deep neural network is used to select the best candidate objects. At the same time, a hierarchical data association strategy is presented to make full use of spatial information and person re-identification (ReID) features to improve tracking performance. Wang et al.^5^ proposed the JDE algorithm. Previous MOT models are all in the “detect first, then extract features” paradigm, but this two-stage execution will decrease efficiency. Hence, JDE does not consider the “separate type” and adopts a “joint type”, which incorporates the appearance embedding model into the detector, so that the model can simultaneously output detection results and ReID information, effectively improving the real-time performance of multi-object tracking. The detection branch of the JDE algorithm is based on the anchor-based approaches, which is not suitable for learning ReID features, so Zhang et al.^6^ described the FairMOT algorithm to solve the “ambiguity” problem caused by anchors in detection and re-identification. This method uses the anchor-free object detection architecture, named CenterNet^7^, as the detection branch. Meanwhile, it applies multi-layer feature fusion to improve the ability of scale transformation and explores the dimensional issue of ReID features, which can solve the “fairness” problem of the detection branch and the re-identification branch and steadily improves the tracking performance. This paper considers the practical application aspects of pedestrian multi-object tracking algorithms, so it chooses FairMOT, based on HRNet and Anchor-free Detection Network, as the basic framework due to its efficiency and robustness.

The contributions of this paper are:

1. HRNet32 is utilized as the baseline to enhance the feature extraction ability, and the Polarized Self-Attention (PSA) attention mechanism is introduced to pay more attention to the pedestrian target and optimize the person re-identification branch.
2. The Circle Loss is employed to train the ReID branch and makes the similar features more compact, the heterogeneous features more distant, and the final extracted ReID features more discriminative.
3. Compared with the existing advanced algorithms on the multi-object tracking dataset MOT17, the accuracy and robustness of the proposed method are effectively improved.

## The proposed network

### Network architecture

**Figure 1:**
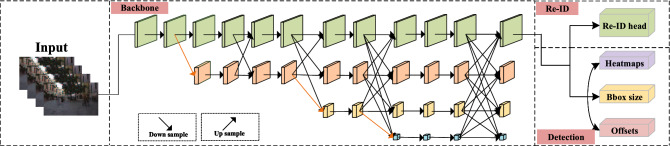
Overall network architecture.

In this paper, considering the higher accuracy required in practical applications, the High-Resolution Net (HRNet)^8^ is adopted as the baseline to achieve better semantic representation and position sensitivity because it can process multiple resolution network branches in parallel and continuously carry out information interaction between different branches. The overall network architecture is illustrated in Fig. 1.

#### HRNet

The purpose of the HRNet is to learn high-resolution representations with strong position sensitivity. Unlike networks, such as AlexNet^9^, VGGNet^10^, GoogleNet^11^, ResNet^12^, and DenseNet^13^, which contain low resolution representations and connect high resolution and low resolution convolution in series, it adopts a “parallel” structure, so that the whole process can maintain high resolution representations and interact with multi-channel resolution features. In addition, the high-resolution map makes it more spatially precise, and the low-resolution map makes it more semantically adequate. Therefore, the final obtained features have high positional sensitivity and strong characterization capability.

The HRNet uses a high-resolution sub-network as the first stage. Then, low-resolution convolutional branches are gradually added and connected in parallel. Thus, the final network consists of several stages, where the nth segment contains n convolutional branches and n different resolutions. Meanwhile, each high-resolution feature representation in the first stage can receive information from its parallel convolution branches, which makes the high-resolution representation more informative and can repeatedly exchange the information from parallel resolution features for multi-resolution fusions. As a result, the output keypoint heatmap is more precise, and has higher spatial resolution accuracy. In this paper, HRNet32 is used as the baseline network.

#### Polarized self-attention

Attention mechanisms are widely used in various deep learning tasks, e.g., image recognition, natural language processing, speech recognition, etc. Attention stems from the study of human vision, and in cognitive science, due to bottlenecks in information processing, humans selectively focus on a portion of all information while ignoring the rest^14^. It enables neural networks to pay more attention to specific and important information from the input.

**Figure 2:**
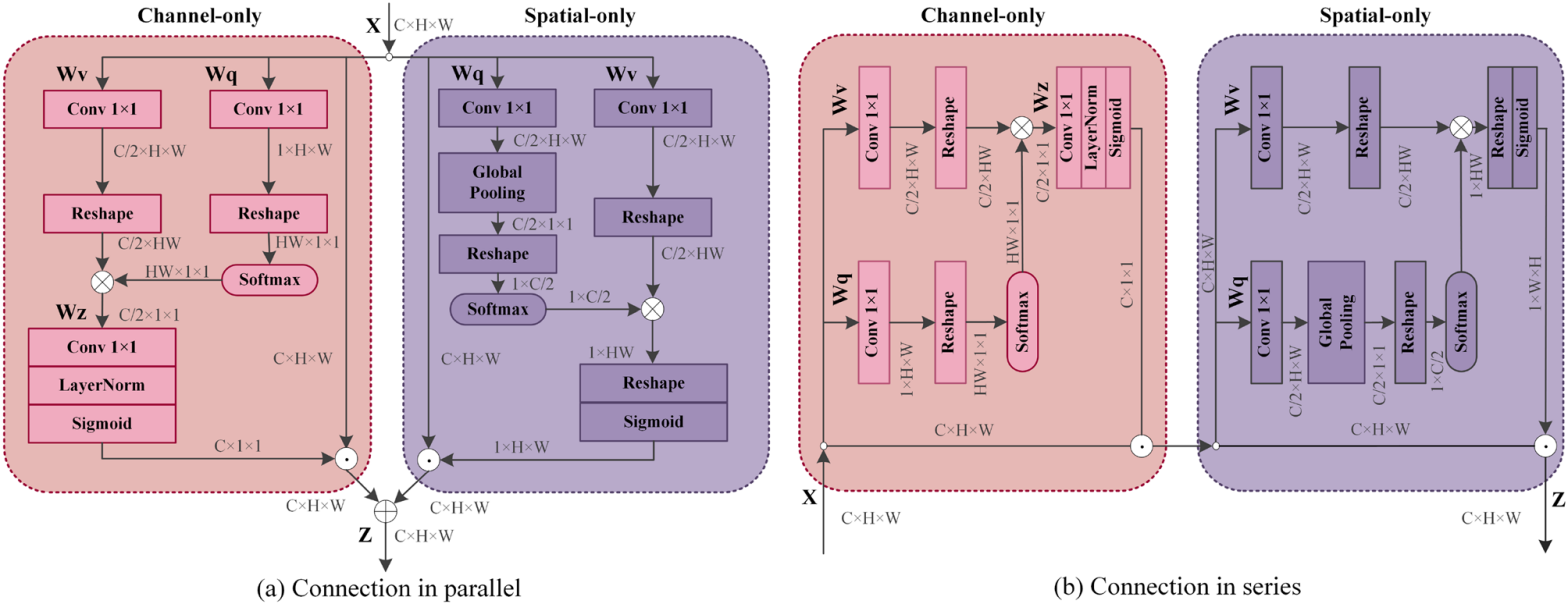
This is The PSA module (**a**) Connection in parallel and (**b**) Connection in series.

In order to improve the performance of the baseline network in our paper, the Polarized Self-Attention (PSA) mechanism^15^ is added to the backbone. Unlike other attention mechanism structures (such as SENet^16^, ECANet^17^, CBAM^18^, etc.), the weights of the PSA mechanism are obtained from the self-attention structure rather than convolutional and fully-connected layers. At the same time, a dimensionality reduction operation is conducted to reduce the calculation cost. The PSA structure is shown in Fig. 2. We can see that PSA does not compress to a great extent in both the spatial and channel dimensions, which makes the information loss relatively small. While previous attention mechanisms used nonlinear functions for probability estimation usually apply either Softmax or Sigmoid, PSA adopts a combination of Softmax and Sigmoid in the channel and spatial branches to increase the nonlinearity to fit a more realistic output distribution.

From Fig. 2, PSA can be divided into two branches: Channel Branch and Space Branch. The implementation of PSA, taking the Channel-only branch as an example, firstly reshapes the input $\mathbf{{X}} \in {R^{{C_{in}} \times H \times W}}$ (input image size $1088\times 608$) into ${W_v}\left( X \right)$ (abbreviated as V) and ${W_q}\left( X \right)$ (abbreviated as Q), respectively, through a $1\times 1$ convolution kernel. Then, the channel dimension of V becomes 1/2 of the original one, and the channel dimension of Q is compressed to 1. Next, Q is multiplied by V after information enhancement by Softmax, and the channel is up-dimensioned by $1\times 1$ convolution. Finally, the parameters are mapped between (0, 1) by the Sigmoid function. The weights of the channel branches are calculated as shown in Eq. (1):

1 $$\begin{aligned} \quad \quad \quad A_{{\textrm{chb}}}\left( X \right) = {F_{SG}}\left[ {{W_{z\mid {\theta _1}}}{\left( {{\sigma _1}\left( {{W_v}\left( X \right) } \right) \times {F_{SM}}\left( {{\sigma _2}\left( {{W_{\textrm{q}}}\left( X \right) } \right) } \right) }\right) }} \right] \end{aligned}$$

where *X* is an input feature tensor, *W* represents a $1\times 1$ convolution operation, $\sigma$ denotes reshape operation, ${F_{SM}}$ stands for Softmax information enhancement, and ${F_{SG}}$ indicates the Sigmoid operation. The equation for calculating the weights of the spatial branches is as follows:

2 $$\begin{aligned} A_{{\textrm{spb}}}\left( X \right) = {F_{SG}}\left[ {{\sigma _3}\left( {{F_{SM}}\left( {{\sigma _1}\left( {{F_{GP}}\left( {{W_q}\left( X \right) } \right) } \right) } \right) \times {\sigma _2}\left( {{W_v}\left( X \right) } \right) } \right) } \right] \end{aligned}$$

${F_{GP}}$ in Eq. (2) represents the global pooling operation. The Spatial-only branch is analyzed, and similarly, the features are first rescaled by a $1\times 1$ convolution operation to obtain Q and V.

Unlike the channel branch, where all channel dimensions become half, the feature Q branch adds a global pooling operation to compress the spatial dimensions from $\hbox {H}\times \hbox {W}$ to $1\times 1$ and through the Softmax operation for information augmentation. Then Q and V are matrix multiplied. Eventually, the Sigmoid function mapped the parameters, making the parameters constrained between (0, 1).

There are two fusion methods in PSA for the two branches mentioned above: parallel and series connections. The specific connections are shown in Fig. 2 and the series and parallel fusion calculation equations are calculated as:

3 $$\begin{aligned} {\textrm{PS}}{{\textrm{A}}_p}\left( X \right)= {Z_{ch}} + {Z_{{\textrm{sp}}}} \end{aligned}$$

4 $$\begin{aligned} {\textrm{PS}}{{\textrm{A}}_s}\left( X \right)= {Z_{sp}}\left( {{Z_{ch}}} \right) \end{aligned}$$

where $PS{A_p}$ and $PS{A_s}$ denote the way to fuse two branches in parallel and in series, respectively. In this paper, experiments are conducted for both fusion methods (as shown in Table 1), and the effects of both fusion methods on our model results are analyzed. The experimental settings are listed in **Implementation** and the meaning of the metrics is described in **Evaluation Metrics**.

**Table 1:** Comparative experimental results on series/parallel fusion approaches of the PSA attention mechanism on the HRNet32 network.

Sequences	Tracks	Density	Model	MOTA$\uparrow$ (%)	IDs$\downarrow$	IDF1$\uparrow$ (%)	IDP (%)	IDR (%)
TUD-Campus	*N*=8	5.1	HRNet32+PSAp	75.8	0	86.5	97.6	77.3
			HRNet32+PSAs	76.6	0	87.1	96.9	79.1
ADL-Rundle-6	*N*=24	9.5	HRNet32+PSAp	73.1	25	69.9	76.0	64.7
			HRNet32+PSAs	73.7	23	75.3	81.7	69.9
ADL-Rundle-8	*N*=28	10.4	HRNet32+PSAp	60.8	23	74.1	76.3	72.0
			HRNet32+PSAs	61.6	19	75.0	77.4	72.7
ETH-Sunnyday	*N*=30	5.2	HRNet32+PSAp	73.7	8	84.6	93.0	77.5
			HRNet32+PSAs	76.0	6	85.7	94.6	78.4
KITTI-13	*N*=42	2.2	HRNet32+PSAp	49.5	18	68.8	81.9	59.2
			HRNet32+PSAs	51.5	14	69.9	84.9	59.5

**Figure 3:**
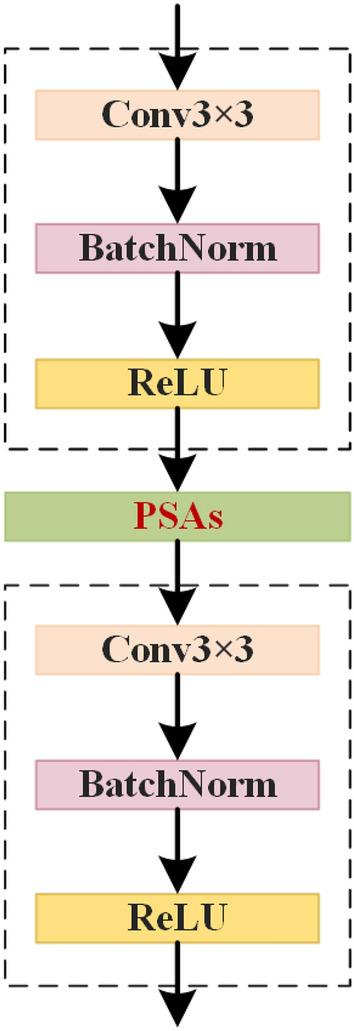
This is PSAs+basic_block.

As shown in Table 1,we randomly selected datasets with different levels of crowd density for comparison experiments. Among them, when the density is 2.2, the series fusion’s MOTA is 2.0% higher, IDF1 is 1.1% higher, and IDs is reduced by 4; when the density is 5.1, the series fusion’s MOTA is 0.8% higher, IDF1 is 0.6% higher. From the experimental results, series fusion is better than parallel fusion. The combined metrics show that the sequential layout of PSAs is a better additive for our baseline network. Therefore, in this paper, PSAs is chosen as the attention mechanism and the plugged position of PSAs is the same as^15^, as shown in Fig. 3.

### Output branches

As shown in Fig. 1, the FairMOT framework outputs detection branches (heatmap, bounding box size, and offset) and the re-identification branch in parallel. Heatmap is in charge of estimating the locations of the target centroid with a dimension of $1\times \hbox {H}\times \hbox {W}$. Bounding box size is in charge of the estimation of the width and height of the target detection box at each anchor point. Offset is the output centroid offset to locate objects more accurately. Both the box offset head and the box size head output dimensions are $2\times \hbox {H}\times \hbox {W}$.

The mathematical expression for the loss function of heatmap is given in Eq. (5), which is defined as a pixel-level logistic regression with focal loss^19^.

5 $$\begin{aligned} {L_{{\textrm{heat}}}} = - \frac{1}{N}\sum \limits _{xy} {\left\{ {\begin{array}{*{20}{l}} {{{\left( {1 - {{\hat{M}}_{xy}}} \right) }^\alpha }log{{\hat{M}}_{xy}}}\\ {{{\left( {1 - {M_{xy}}} \right) }^\beta }{{\left( {{{\hat{M}}_{xy}}} \right) }^\alpha }log\left( {1 - {{\hat{M}}_{xy}}} \right) } \end{array}\begin{array}{*{20}{l}} {\begin{array}{*{20}{c}} {}&{}{{M_{xy}} = 1} \end{array}}\\ {\begin{array}{*{20}{c}} {}&{}{otherwise} \end{array}} \end{array}} \right. } \end{aligned}$$

where *N* denotes the amount of target pedestrians, ${M_{xy}}$ is the heatmap response at (x, y), and ${\hat{M}_{xy}}$ is the estimated heatmap response at (x, y). $\alpha$ and $\beta$ are the predetermined parameters, as set in Centernet^7^, with $\alpha =2$ and $\beta =4$.

The loss functions for offset and bounding box size are represented by $l_{1}$ losses:

6 $$\begin{aligned} {L_{box}} = \sum \limits _{i = 1}^N {\mathop {\left\| {{o^i} - {{\hat{o}}^i}} \right\| }\nolimits _1 } + 0.1\mathop {\left\| {{s^i} - {{\hat{s}}^i}} \right\| }\nolimits _1 \end{aligned}$$

where ${O^i}$ indicates the ground-truth offset, ${\hat{O}^i}$ denotes the estimated offset, ${S^i}$ represents the size of the ground truth box, and ${\hat{S}^i}$ refers to the estimated bounding box size. Meanwhile, ${S^i}$ and ${O^i}$ are calculated as:

7 $$\begin{aligned} \left\{ {\begin{array}{*{20}{l}} {{S^i} = \left( {x_2^i - x_1^i{\textrm{,y}}_2^i - y_1^i} \right) }\\ {{O^i} = \left( {\frac{{c_x^i}}{4},\frac{{c_{\textrm{y}}^i}}{4}} \right) - \left( {\left\lfloor {\frac{{c_x^i}}{4}} \right\rfloor ,\left\lfloor {\frac{{c_y^i}}{4}} \right\rfloor } \right) } \end{array}} \right. \ \end{aligned}$$

where $\left( {x_1^i,y_1^i,x_2^i,y_2^i} \right)$ are the coordinates of each ground truth box, and *c* denotes the object center.

The objective of the re-identification branch is to generate discriminative features and extract target depth features based on the location response of object detection. The ideal situation is that the distance between the same identity is less than the distance between different identities. Toward this objective, a convolutional layer of 128 kernels is used here to characterize the depth epigenetic features of target objects. The mathematical expression of the loss function of the original ReID branch is as:

8 $$\begin{aligned} {L_{{\textrm{identity}}}} = - \sum \limits _{i = 1}^N {\sum \limits _{{\textrm{k}} = 1}^K {{L^i}\left( {\textrm{k}} \right) log\left( {p\left( k \right) } \right) } } \end{aligned}$$

where K represents the number of target categories. Thus, the total loss function can be written as:

9 $$\begin{aligned} \begin{aligned} {L_{total}}&= \frac{1}{2}\left( {\frac{1}{{{e^{{w_1}}}}}{L_{detection}} + \frac{1}{{{e^{{w_2}}}}}{L_{identity}} + {w_1} + {w_2}} \right) \\&= \frac{1}{2}\left( {\frac{1}{{{e^{{w_1}}}}}\left( {{L_{heat}} + {L_{box}}} \right) + \frac{1}{{{e^{{w_2}}}}}{L_{identity}} + {w_1} + {w_2}} \right) \\ \end{aligned} \end{aligned}$$

where ${w_1}$ is the weight of the object detection branch, and ${w_2}$ is the weight of the re-identification branch.

### ReID loss improvements

The main role of the person re-identification branch is to extract highly-discriminative features. In this section, the optimization of the loss function is described.

**Figure 4:**
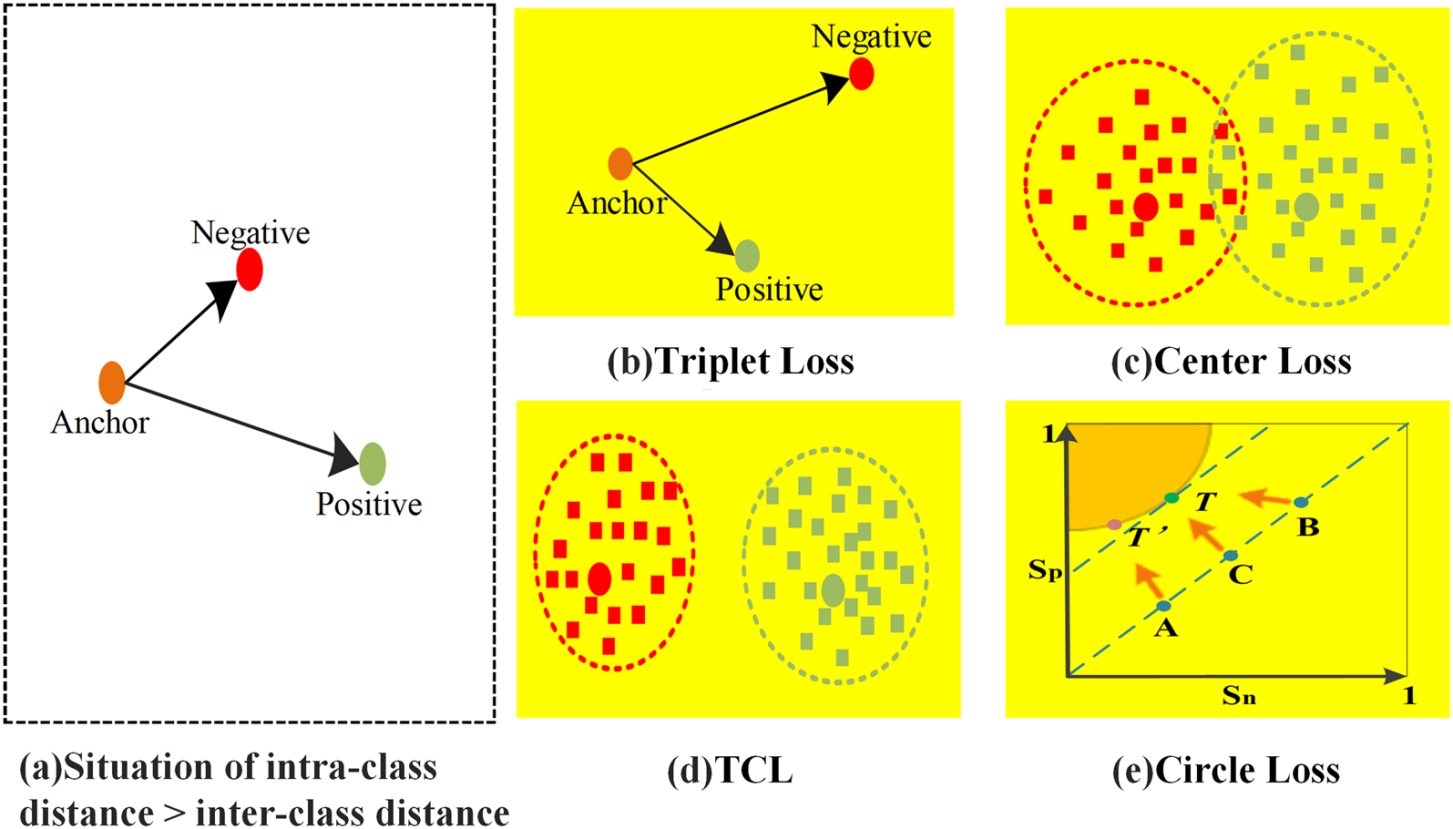
This is Features of different loss functions.

The Cross Entropy (CE) loss is commonly used in person re-identification tasks and is essential to retrieving objects, whose requirement is that the distance of within-class features to be smaller than the distance of between-class features. However, the CE loss function only focuses on the correctness of the classification and does not consider the clustering requirements between features. Therefore, the triplet loss function^20^, which sets the input into three groups: anchor, positive sample, and negative sample, is proposed to solve this problem by adding constraints to minimize the intra-class distance and increase the inter-class distance while satisfying the condition that the intra-class distance is smaller than the inter-class distance. However, triplet loss learns the relative distance between samples rather than the absolute distance, which does not consider the intra-class compactness. Then, Center Loss^21^ is proposed to further reduce the intra-class distance by learning the center of each class, which makes the clustering of homogeneous samples more significant. Nevertheless, Center Loss focuses on clustering same-class sample features, so it is generally used as an auxiliary loss function. Accordingly, an improved version of the loss function Triplet Center Loss (TCL) is proposed in^22^, which combines the advantages of Triplet Loss and Center Loss to minimize the intra-class distance and maximize the inter-class distance of depth features. Besides, most loss functions have a uniform margin for intra-class similarity and inter-class similarity, which makes the distinction between positive and negative samples insufficient and the optimization inflexible at the time of model convergence. Hence, Sun et al.^23^ proposed the Circle Loss. Unlike most loss functions, it formally unifies the loss functions under the two basic learning paradigms (classification learning and pairwise learning) from the unified similarity pair optimization perspective. It has high optimization flexibility, and each similarity score can be learned at a different gradient with a clear convergence target (as shown in Fig. 4e) so that to facilitate the learning of deep features. A visual representation of the features for each loss function listed above is shown in Fig. 4.

In order to demonstrate the contribution of each loss function, a comparison experiment is conducted in this paper. The comparative results are presented in Table 2, and the specific experimental parameters are set as in **Implementation**.

**Table 2:** Impact of different loss functions.

Model	MOTA$\uparrow$ (%)	IDs$\downarrow$	IDF1$\uparrow$ (%)	Rcll$\uparrow$ (%)
TripletCenter Loss	77.6	62	80.9	82.3
CE Loss	78.4	67	80.0	84.7
Circle Loss	78.7	59	79.8	83.7

**Figure 5:**
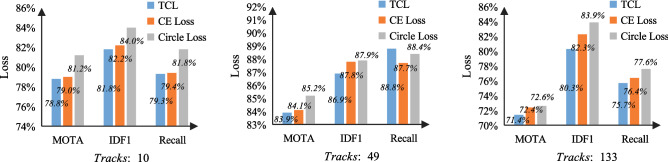
Impact of three loss functions on samples with different number of tracks.

**Figure 6:**
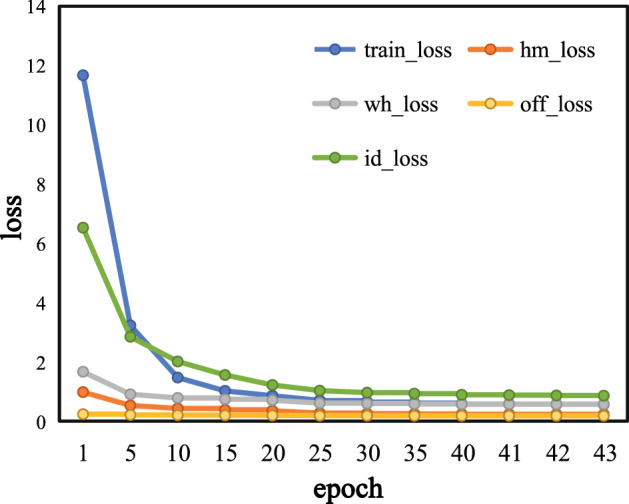
Loss variation during training.

It can be seen from Table 2 that Circle Loss’s MOTA reaches 78.7%, and IDs is 59. In addition, we randomly select samples with different number of tracks for further experimental evaluation (as shown in Fig. 5). According to that, Circle Loss is more effective. The loss variation during the training process is shown in Fig. 6, which includes trainning loss (train_loss), heatmap loss (hm_loss), offset loss (off_loss), box size loss (wh_loss), and identity embedding loss (id_loss). Therefore, Circle Loss is adopted as the loss function of the person re-identification branch, which effectively allows maximizing the intra-class similarity of depth features and minimizing the inter-class similarity. The expression of the unified loss function is shown as:

10 $$\begin{aligned} \begin{aligned} {L_{{\textrm{circle}}}}&= log\left[ {1 + \sum \limits _{i = 1}^K {\sum \limits _{j = 1}^L {exp\left( {\gamma \left( {\alpha _n^js_n^j - \alpha _p^is_p^i} \right) } \right) } } } \right] \\&= log\left[ {1 + \sum \limits _{{\textrm{j}} = 1}^L {exp\left( {\gamma \alpha _n^js_n^j} \right) \sum \limits _{{\textrm{i}} = 1}^K {exp\left( { - \gamma \alpha _p^is_p^i} \right) } } } \right] \\ \end{aligned} \end{aligned}$$

where $s_n^j$ is inter-class similarity, and $s_p^i$ is intra-class similarity. L means there are L inter-class similarity scores and K means there are K intra-class similarity scores. $\gamma$ denotes the scale change parameter, and $a_n^j$ and $a_p^i$ are non-negative weight parameters that constrain the gradient of ${s_n}$ and ${s_p}$ , respectively. By introducing adaptive weighting of hyperparameters $a_n^j$ and $a_p^i$ , the loss function optimization is more flexible. Here, the mathematical definitions of $a_n^j$ and $a_p^i$ are as follows:

11 $$\begin{aligned} \left\{ {\begin{array}{*{20}{c}} {\alpha _p^i} ={{[{{O_p} -s_p^i}]_ +}}\\ {\alpha _n^j} ={{[{{s_n^j} -O_n}]_ +}}\\ \end{array}} \right. \end{aligned}$$

where ${O_p}$ and ${O_n}$ are two hyperparameters as the optimization objectives of ${S_p}$ and ${S_n}$, respectively. Learning from Eq. (11), it is clear that 1) the closer the similarity score is to the optimum, the less weight is given to it; 2) the further it is from the optimum, the more weight is given to it. ${\left[ \cdot \right] _ + }$ indicates that only positive values are taken, which is equivalent to the ReLU operation, and loss will no longer be optimized when the value is less than 0. Because the addition of weighting factors to the loss function results in asymmetry between ${S_p}$ and ${S_n}$, the optimization objective will no longer be adjusted using a uniform margin. Therefore, hyperparameters ${\Delta _{\textrm{n}}}$ and ${\Delta _{\textrm{p}}}$ are also introduced. And eventually, the Circle Loss can be expressed by:

12 $$\begin{aligned} {L_{{\textrm{circle}}}} = log[{1 + \sum \limits _{j = 1}^L {exp\left( {\gamma \alpha _n^j\left( {s_n^j - {\Delta _n}} \right) } \right) } \sum \limits _{i = 1}^K {exp\left( { - \gamma \alpha _p^i\left( {s_p^i - {\Delta _p}} \right) } \right) } }] \end{aligned}$$

where ${\Delta _{\textrm{n}}}$ and ${\Delta _{\textrm{p}}}$ are the inter-class and intra-class margins, respectively.

### Data association strategy

**Figure 7:**
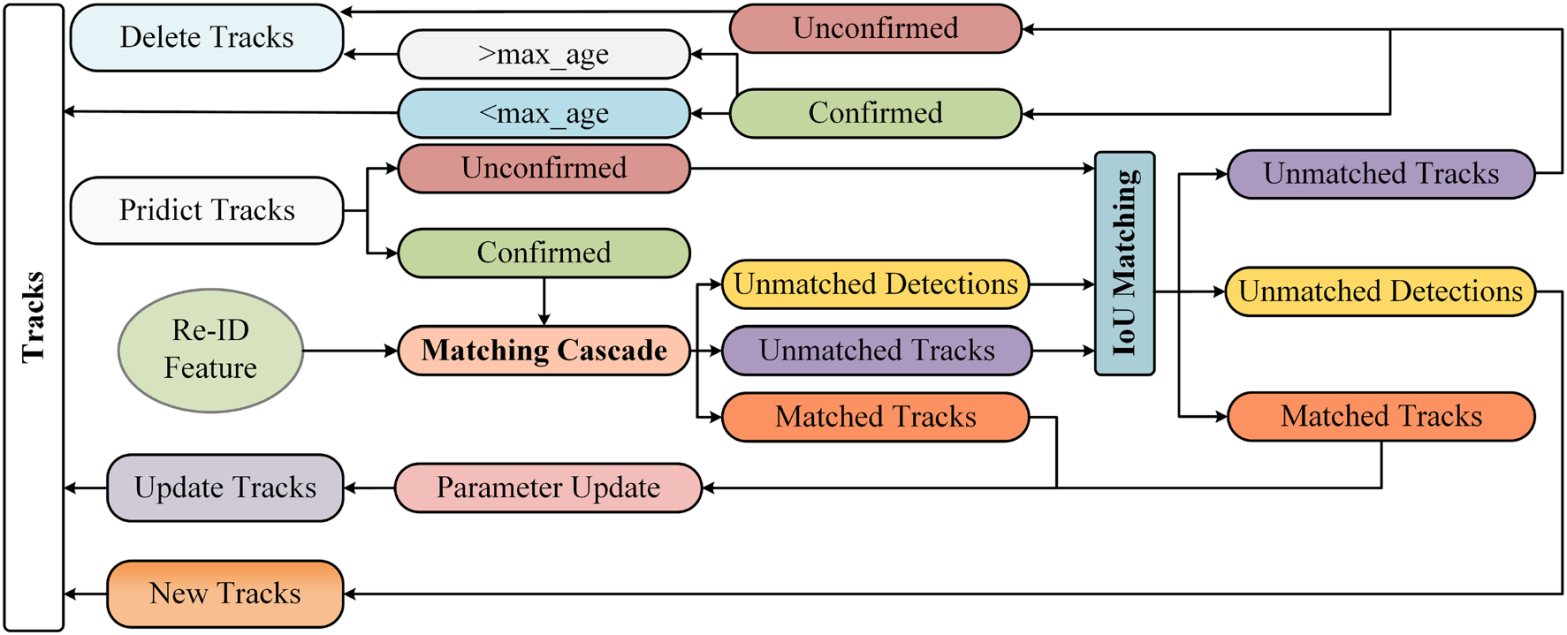
This is Tracking framework.

The data association section is presented in Fig. 7. The extracted depth feature information is matched in cascade with the ReID information on the determined trajectory predicted by Kalman filters, and the preliminary data association results can be obtained: matched tracks, unmatched detections, and unmatched trajectories. Hungarian matches^24^ are then performed for unmatched tracks, unmatched detections, and uncertain trajectories predicted by Kalman filters, and the matched tractories are fed into the Kalman filter for updating. The unmatched detection result is initialized as a new trajectory, designated as deterministic if it can match the object in three consecutive frames. It is subsequently fed into the Kalman filter for parameter updating. For unmatched but definite trajectory, if the number of consecutive mismatched frames is less than max_age, it is added to the tracking sequence; Otherwise, the trajectory is deleted. For the case of unmatched and unconfirmed trajectory, the trajectory can be deleted directly.

## Experimental results and analysis

### Datasets

The CUHK-SYSU dataset is the inaugural massive pedestrian retrieval dataset published by Wang et al.^25^ from the Chinese University of Hong Kong. It contains 18184 images with 96143 pedestrians, including 8432 identities. The dataset consists of a training set and a test set, where the training set contains 11206 images with a total of 55272 pedestrians (5523 identities), and the test set contains 6978 images with a total of 40781 pedestrians (2900 identities).

MOTChallenge is a large public dataset for pedestrian multi-object tracking, containing MOT15, MOT16, MOT17, and MOT20 datasets. Among them, the MOT15^26^ dataset consists of 11 sequences for training and 11 for testing, with a total of 11286 frames or 996 seconds of video^27^. The MOT16^28^ dataset contains 14 challenging video sequences, half of which are used as the training set and half as the test set, with 11235 frames. The MOT17 dataset, consisting of 14 video sequences where 7 as training sequences with 5136 images and 7 as test sequences with 5919 images, simultaneously provides detection results for three common detectors (DPM, FRCNN, and SDP) for each video sequence. The MOT20^29^ dataset contains a series of 8 videos (4 as the training set and 4 as the test set) with a total of 13410 frames. It is mainly oriented towards dense scenes, with an average crowd density that can reach 246 pedestrians per frame.

### Implementation

The experimental environment is based on an Ubuntu 18.04 LTS operating system, using Python 3.8 and Pytorch 1.7 deep learning framework, occupying 3 NVIDIA GeForce RTX 3090 GPUs. The training set data of CUHKSYSU, PRW^30^, and MOT16 datasets are selected as the training set as well as the MOT15 dataset is used as the validation set during the training process. Considering the comprehensive performance of the model, an Adam optimizer is applied to train the model for 43 epochs (as is shown in Fig. 6). The batch size is set to be 16. Finally, a comparative experiment with other advanced algorithms is carried out on the test set of MOT17.

### Evaluation metrics

In order to make the evaluation of the model more objective and accurate, as well as to make a reasonable comparison with other algorithms, a general evaluation system^31,32^ is conducted in the field of multi-object tracking, and the main metrics are: Multi-object Tracking Accuracy (MOTA), Identification F1 Score (IDF1), Identity switches (IDs), Mostly tracked (MT), Mostly Lost (ML), Identification precision (IDP), Identification Recall (IDR), the number of false positives in the whole video (FP), and the number of false negatives in the whole video (FN)^33^. Among them, MOTA and IDF1 are defined as follows:

13 $$\begin{aligned} MOTA= & {} 1 - \frac{{\sum \nolimits _t {f{n_t} + f{p_t} + id{s_t}} }}{{\sum \nolimits _t {g{t_t}} }} \end{aligned}$$

14 $$\begin{aligned} IDF1= & {} \frac{{IDTP}}{{IDTP + 0.5IDFP + 0.5IDFN}} \end{aligned}$$

### Experimental results

#### Ablation experiments

In this paper, ablation experiments are implemented on subsets of the MOT15 training set (KITTI-13, KITTI-17, ADL-Rundle-6, PETS09-S2L1, TUD-Campus, and TUD-Stadtmitte). Improvement 1 (Im 1) represents the baseline network of HRNet, Improvement 2 (Im 2) is the addition of the polarized attention mechanism PSA, and Improvement 3 (Im 3) is the adoption of Circle Loss to optimize the re-identification branch. The experimental results are shown in Table 3 ($\uparrow$ means the higher the better and $\downarrow$ means the lower the better).

**Table 3:** Model ablation experiments on subsets of MOT15’s training sets.

Im1	Im2	Im3	MOTA$\uparrow$ (%)	IDs$\downarrow$	IDF1$\uparrow$ (%)	IDP (%)	IDR (%)	Rcll (%)
$\checkmark$			77.0	72	77.8	83.5	72.9	82.4
$\checkmark$	$\checkmark$		77.6	62	80.9	87.2	75.4	82.3
$\checkmark$	$\checkmark$	$\checkmark$	78.7	59	79.8	85.2	75.1	83.7

**Figure 8:**
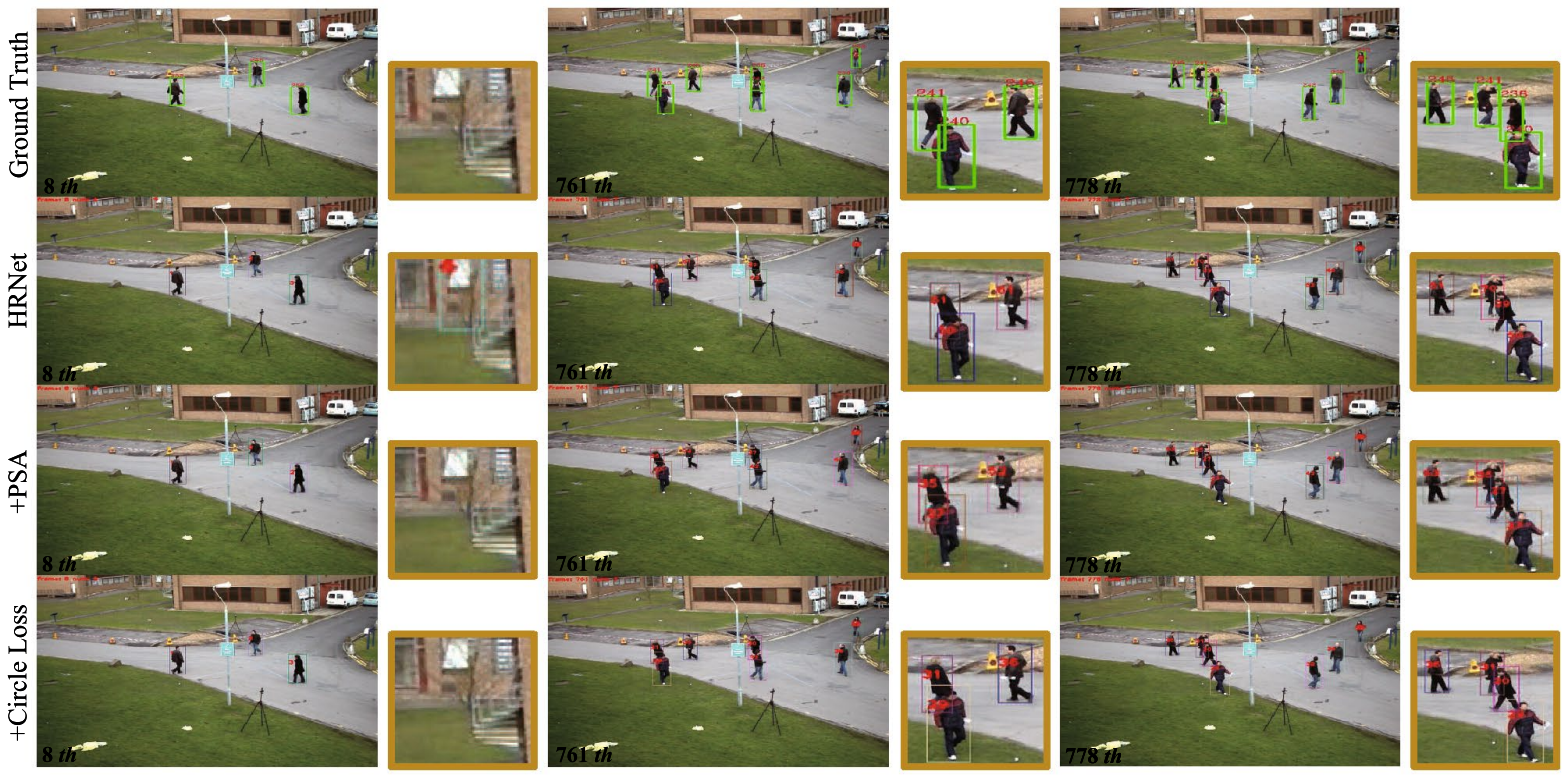
This is Visualization of ablation experiment results.

To make the comparison more intuitive, Fig. 8 visualizes the results of the PETS09-S2L1 dataset. As can be observed from the graph, model based on HRNet32 as the baseline network has a false detection at Frame 8, incorrectly detecting a “non-pedestrian” as the target. At the same time, the pedestrian identified as 51 and the pedestrian identified as 61 in Frame 761 are walking in opposite directions, and their identities are switched in Frame 778. After the PSA attention mechanism is introduced, it can be found that the misdetection has been resolved. But two objects moving in opposite directions, only one can maintain a stable ID while the other’s ID still changes. Thus, the ID switching problem still remains unresolved. Then, the re-identification branch is optimized, and the ID switching phenomenon disappears after introducing Circle Loss. Hence, it can intuitively reflect the correctness of the improved algorithm.

#### Comparative experiments

**Figure 9:**
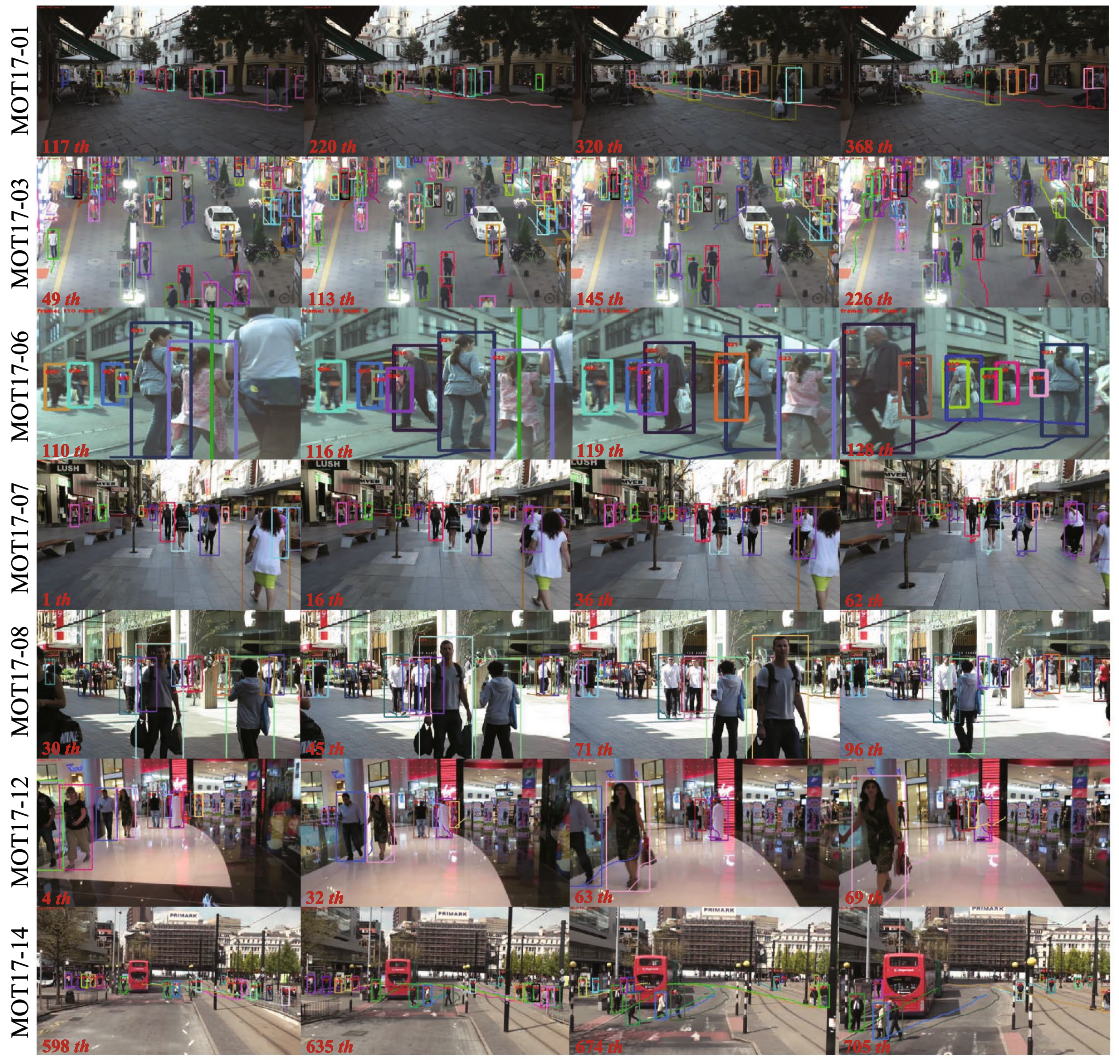
Visualization of tracking trajectory.

For further demonstrating the validity of the algorithm presented in this paper, a comparison with existing mainstream advanced algorithms is proposed on the test set of the MOT17 dataset. The experimental results are shown in Table 4, and the comparative analysis is performed according to the evaluation metrics. Also, the tracking trajectory is shown in Fig. 9.

**Table 4:** Comparison of the proposed algorithm with existing methods.

Model	MOTA$\uparrow$ (%)	IDF1$\uparrow$ (%)	MT$\uparrow$ (%)	ML$\downarrow$ (%)	FP$\downarrow$	FN$\downarrow$	IDs$\downarrow$
DAN^34^	52.4	49.5	21.4	30.7	25,423	2,34,529	8431
Tracktor+CTdet^35^	54.4	56.1	25.7	29.8	44,109	2,10,774	2574
TubeTK^36^	63.0	58.6	31.2	19.9	27,060	1,77,483	4137
CTracker^37^	66.6	57.4	32.2	24.2	22,284	1,60,491	5529
CenterTrack^38^	67.3	59.5	34.9	24.8	23,031	1,58,676	2898
TraDes^39^	69.1	63.9	36.4	21.5	20,892	1,50,060	3555
ByteTrack^40^	80.3	77.3	53.2	14.5	25,491	83,721	2196
Ours	69.5	70.0	35.3	24.3	22,470	1,47,339	2919

From the Table 4, we can see that the MOTA of our model is able to achieve 69.5% on the MOT17 dataset, which is 17.1%, 15.1%, 6.5%, 2.9%, 2.2%, and 0.4% higher than DAN, Tracktor+CTdet, TubeTK, CTracker, CenterTrack, and TraDes algorithms, respectively. Meanwhile, IDF1 can reach 70.0%, FP is reduced by 3021 compared to the ByteTrack algorithm, and the number of identity switches is 2919, which is 5512, 1218, 2610, and 636 times less than that of DAN, TubeTK, CTracker, and TraDes algorithms, respectively. According to the results obtained from the experiments, the advantages of the proposed algorithm in this paper are demonstrated.

## Conclusions

This paper proposed a multi-object tracking algorithm based on FairMOT and Circle Loss. First, HRNet32 was adopted as the baseline network, then polarized attention mechanism PSA was added to improve the performance, and eventually, Circle Loss was selected to optimize the re-identification branch. Compared with existing advanced multi-object tracking algorithms, the proposed method achieved higher multi-object tracking accuracy and a better ability to maintain a stable pedestrian identity.

### Informed consent

For online open-access publication of the images has been obtained from all the participants.

## Data Availability

The data that support the findings of this study are available from the corresponding author, Yuting He, upon reasonable request.
